# Impact of Nursing Professionalism on Perception of Patient Privacy Protection in Nursing Students: Mediating Effect of Nursing Informatics Competency

**DOI:** 10.3390/healthcare9101364

**Published:** 2021-10-14

**Authors:** Hyun-Kyeong Park, Yeo-Won Jeong

**Affiliations:** 1 Department of Nursing, Graduate School, Dongguk University, Gyeongju 38066, Korea; sg3679@naver.com; 2 Department of Nursing, College of Nursing, Dongguk University, Gyeongju 38066, Korea

**Keywords:** professionalism, student, nursing, informatics, patient information

## Abstract

In recent times, as the healthcare system becomes more informational, the importance of patient privacy protection increases, making it necessary to identify factors that affect the perception of patient privacy protection. This study aimed to evaluate the relationship between nursing professionalism and the perception of patient privacy protection and the mediating role of nursing informatics competency. The study recruited 242 nursing students who had experienced dealing with patient information during clinical practice. The mediating model using the Hayes’ PROCESS macro (Model 4) was employed to test the study hypothesis. Nursing professionalism was found to be positively and significantly associated with the perception of patient privacy protection (*β* = 0.09, *p* = 0.021) with the mediation of nursing informatics (*β* = 0.18, *p* < 0.001). Our findings showed that nursing professionalism and nursing informatics competency determined the perception of patient privacy protection. The mediating role of nursing informatics competency implies that curricula designed to enhance nursing informatics competency of nursing students may increase their perception of patient privacy protection.

## 1. Introduction

The current healthcare system collects and stores the personal information of numerous patients in line with the Information Age, helping medical professionals to conveniently provide medical care to patients in an efficient manner. Thus, medical information related to patient privacy protection has emerged as an important issue in information exchange [1]. Personal privacy contains extremely sensitive information like medical treatment details and health information and should be handled with care, as there is a high possibility of invasion of privacy if leaked [2]. As the social demand for patient privacy protection increases, the WHO emphasized that efforts to protect patient privacy should be a top priority in all medical institutions as patients’ information becomes digitized [3]. Since nurses, in particular, frequently access personal information while being in contact with patients every day for a prolonged period, they are obliged to fulfill their ethical and legal responsibilities in protecting their medical information to respect such personal information and maintain trust [4,5]. Nursing students, as prospective nurses, also deal with patient privacy directly during practice and collect patient information while often sharing that information with other students [6]. Since trainees at medical colleges handle patient medical information only during the training period, problems regarding patient privacy leakage are being raised as they have less responsibility for information protection compared to other hospital staff, and are not familiar with complementary methods or procedures [7,8]. They also have low awareness regarding patient privacy. According to a study by Cannon and Caldwell [9], there have been cases in which nursing students received a warning from the hospital for sharing patient privacy and family relationships through informal discussions, but they did not consider this to be a leak of sensitive patient privacy. Although awareness of patient privacy protection needs to be raised in nursing students who directly deal with it, studies on the level of patient privacy protection and influencing factors in nursing students are insufficient [2]. The leakage of patient privacy can damage the professionalism and reputation of a medical institution and may result in violating many ethical and legal regulations that can undermine the trust of other institutions and the public [10]. As the importance of patient privacy protection in the medical field has become increasingly emphasized, it is necessary to study the factors affecting the protection of patient privacy by nursing students, who are prospective nurses.

### Perception of Patient Privacy Protection, Nursing Professionalism, and the Mediating Effect of Nursing Informatics Competency

Patient personal information refers to all patient-related medical information collected during the treatment process, such as name, resident registration number, contact information, patient’s health condition, physical characteristics, medical history (including family history), physical strength, genetic information, and patient registration number [11]. The patient reserves the right to share his/her personal information, which is guaranteed by the Personal Information Protection Act. Patient privacy protection in medical institutions is a professional responsibility for all healthcare professionals, especially nurses, as well as a key concept in nursing ethics [4,5,6,7,8,9,10,11,12]. Additionally, as nursing students have more opportunities to access patients’ medical and personal information, they are required to take responsibility for patient privacy protection and improve their professionalism.

Nursing professionalism refers to a professional and systematic view of nursing as a profession [13]. Schmidt and MacArthur mentioned that patient privacy protection is one of the methods that can measure the value of the nursing profession [14]. A previous study found a significant positive correlation between the patient’s perception of medical information protection and the professionalism of nursing students [5]. As the opportunities for leakage of patient privacy increase due to the use of online learning, social media, and email correspondence among nursing students, the need to fulfill professional and ethical obligations for patient privacy protection by establishing desirable nursing professionalism is the pressing priority [15].

Along with patient privacy protection, one of the most important roles of nurses is to collect and interpret data to provide safe and effective patient care [16], and in the present times, informatics is one of the core competencies that healthcare professionals should accomplish [17]. Nursing informatics competency refers to the knowledge, skills, and attitude of using various nursing, computer, and information sciences [18]. In medical setups, informatics technology is used in important decision-making for optimal patient outcomes [19], along with in collecting, managing, and protecting patient privacy. The higher the nursing informatics competency of nursing students, the higher their awareness of patient privacy protection [8,9,10,11,12,13,14,15,16,17,18,19,20]. It has been reported that insufficient nursing competency can lead to the inability to properly use electronic patient privacy for nursing and research, resulting in problems in patient privacy protection. Furthermore, according to a study, nursing informatics competency includes the concept of one’s role as a nurse in addition to basic computer knowledge and skills [21,22]. The need to develop nursing informatics competency is emerging as a form of nursing professionalism in recent times to protect patient privacy according to medical informatization [23]. From this perspective, a significant positive relationship between nursing informatics competency and nursing professionalism is expected to be implemented among the nursing staff. Nursing informatics competency is expected to have a mediating effect on the relationship between nursing professionalism and the perception of patient privacy protection. However, no related studies have yet been conducted on this topic.

Thus, this study aimed to examine the level of nursing professionalism, nursing informatics, and perception of patient privacy protection, and the mediating effect of nursing informatics competency on the relationship between nursing professionalism and perception of patient privacy protection. The proposed hypotheses were as follows: H1 Nursing professionalism is positively associated with the perception of patient privacy protection of nursing students, and H2 Nursing informatics competency has mediating effects on the relationship between nursing professionalism and perception of patient privacy protection.

## 2. Materials and Methods

### 2.1. Study Design

This study used a cross-sectional design to investigate the mediating effect of nursing informatics competency on the relationship between nursing professionalism and perception of patient privacy protection.

### 2.2. Participants

This study used a cross-sectional design. A total of 242 Korean nursing students (213 females and 29 males) in their 3rd and 4th year who had direct access to patient privacy during their practice were recruited from two universities located in B and G cities. They understood the purpose of the study and agreed to participate. The sample size was calculated using the G*Power 3.1.9.2 program (Heinreich-Heine-Universität, Düsseldorf, Germany) to evaluate the effects on patient privacy information perception for regression analysis. The minimum number of participants was 178 to facilitate a statistical power of 0.95 at a significance level of 0.05, and a median effect size of 0.15, with a conservative effect size with the number of predictors of 11. We recruited a total of 250 participants, considering a 20% dropout rate of the online survey; 242 valid completed questionnaires were used for the final analysis.

### 2.3. Measures

#### 2.3.1. Nursing Professionalism

Nursing professionalism was assessed using a scale developed by Yeun et al. [13]. There was a total of 26 items in five domains: self-concept of the profession, social awareness, professionalism of nursing, the role of nursing service, and originality of nursing. The responses were recorded using a 5-point Likert scale (1 = not at all to 5 = a lot), where a higher score indicated higher nursing professionalism. The total Cronbach’s alpha for this study was 0.89.

#### 2.3.2. Nursing Informatics Competency

To measure nursing informatics competency, the Korean version of the Nursing Informatics Competency Scale [24] developed by Stagger [25] was used. A total of 25 questionnaires covered three domains: computer technology, information technology, and information knowledge. The responses were recorded using a 5-point Likert scale (1 = not at all, 5 = a lot), where a higher total score indicated a higher level of nursing informatics competency. The Cronbach’s alpha for this study was 0.87.

#### 2.3.3. Perception of Patient Privacy Protection

Perception of patient privacy protection of nursing students was assessed using a modified scale [7], originally developed by Lee and Park [26]. The scale is divided into two parts: patient privacy awareness and practice; only the awareness field was used in this study. This scale consists of 23 items in three domains: communication, system integration, and patient information management. The responses were recorded on a 5-point Likert scale (1 = not at all to 5 = a lot), where the higher total score indicated a higher patient’s privacy protection. The Cronbach’s α was 0.90.

### 2.4. Covariates

The covariates included age, gender, grade, religion, perception of the need for patient privacy protection-related education, and education on patient privacy protection. The perception of the nursing students regarding the need for patient privacy protection-related education was assessed by the question, “Do you think that the patient privacy protection related education is needed?”. Regarding the education of patient privacy protection, the question, “Have you ever been formally educated on patient privacy protection?” was asked since the curriculum of patient privacy protection for nursing students before clinical practice is not compulsory by the university or clinical practice hospital. Participants who answered ‘yes’ to the first question were asked additional questions on the number of education sessions, location, and time.

### 2.5. Procedure and Ethical Consideration

The data retrieved for this study were collected via a self-reported online survey distributed among undergraduate nursing students between October and November 2020 after obtaining approval from the Dongguk University Institutional Review Board (IRB) (DGU IRB 2000029) for ethical protection of the participants in the study.

The structured questionnaire was uploaded on a website where the researcher could sign up as a nursing student. The first page of the survey carried the necessity and purpose of the study, data collection method, etc., and a button (“I agree”) which upon clicking, would denote that the participant has agreed to participate in the survey.

It took about 10–15 min per person to fill out the questionnaire, and those who did not understand a question could contact the researcher who would help them understand it.

On the completion of the data collection, gifts worth 5000 won were presented to the participants who responded to and completed the questionnaire.

### 2.6. Data Analysis

The data were analyzed using SPSS/WIN 25.0 (IBM Corp, Armonk, NY, USA) and SPSS PROCESS macro, Version 3.4. The general characteristics and main variables were analyzed using descriptive statistics. Means and standard deviations were used for continuous variables, and absolute (n) and relative frequencies (%) were used for categorical variables. Correlations with the main variables were processed using Pearson’s correlation coefficient. The PROCESS macro for SPSS (Model 4) was used to examine the association between nursing professionalism and patient privacy protection and the mediating effect of nursing informatics competency (Hayes, 2012, 2017). The 95% bias-corrected confidence interval from 5000 resamples was generated using the bias-corrected bootstrapping method. The number of bootstrapping sizes was 5000. Significant indirect effects were identified as *p* < 0.05 when the confidence interval (CI) did not include zero.

## 3. Results

### 3.1. General Characteristics and Education of Patient Privacy Protection

A total of 242 participants participated in the study of which 62.4% (151) were 4th year students (seniors). Of the 242, 221 participants responded that patient privacy protection-related education was necessary; of the total, 57.4% had reported having received patient privacy protection education (Table 1).

### 3.2. Descriptive Statistics and Correlation among Main Variables

The mean scores of nursing professionalism, nursing informatics competency, and perception of patient privacy protection were 105.28 ± 12.86, 92.76 ± 11.34, and 99.16 ± 9.39, respectively (Table 2). Perception of patient privacy protection was positively correlated with nursing professionalism (*r* = 0.307, *p* < 0.001) and nursing informatics competency (*r* = 0.289, *p* < 0.001). Nursing professionalism was found to be significantly correlated with nursing informatics competency (*r* = 0.236, *p* < 0.001).

### 3.3. Mediating Effect of Nursing Informatics Competency on the Relationship between Nursing Professionalism and Perception of Patient Privacy Protection

Regarding hypothesis 1 of the study, nursing professionalism displayed a significant direct effect on patient privacy protection (*B* = 0.09, *p* = 0.021) after adjusting for potential covariates (Table 3). Thus, Hypothesis 1 is supported.

**Figure 1:**
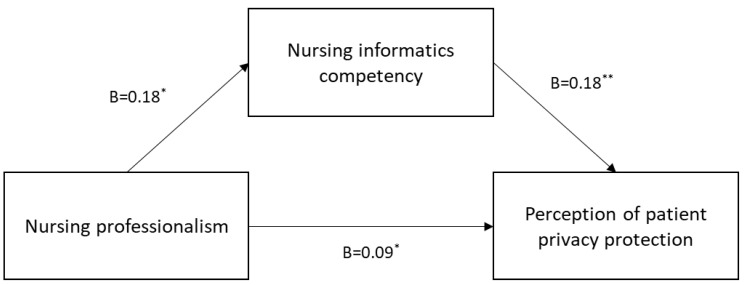
Mediating effect of nursing informatics competency on the relationship between nursing professionalism and perception of patient privacy protection (* *p* < 0.05, ** *p* < 0.001).

As for Hypothesis 2, Table 3 and Figure 1 show the mediating effect of nursing informatics competency. The findings showed a significantly positive effect of nursing professionalism on nursing informatics competency (*B* = 0.18, *p* = 0.001). Furthermore, a significant positive effect of nursing informatics competency was also observed on patient privacy protection (*B* = 0.18, *p* < 0.001). Thus, nursing informatics competency showed a partially mediated effect on the relationship between nursing professionalism and patient privacy protection. The indirect impact of nursing informatics competency was significant for the relationship between nursing professionalism and patient privacy protection, as calculated by the bootstrapping method (*B* = 0.03, standard error = 0.01, BootLLCI, BootULCI = 0.00, 0.06).

## 4. Discussion

The findings of the study support the hypotheses that the concept of nursing professionalism has a positive association with perception of patient privacy protection and that nursing informatics competency has a mediating effect on this relationship. This study is the first to evaluate the association between nursing professionalism and informatics competency, and the mediating effect of nursing informatics competency on the relationship between nursing professionalism and perception of patient privacy protection.

Consistent with hypothesis H1, nursing students in this study who reported higher scores for nursing professionalism displayed an increased awareness of patient privacy protection. This is consistent with previous findings. Nursing professionalism affects the perception of patient privacy protection, such as protection of patients’ privacy and personal information [5,6,7,8,9,10,11,12,13,14,15,16,17,18,19,20,21,22,23,24,25,26,27]. The findings confirmed that the higher the nursing professionalism, the higher the protection of patient privacy [28]. Nursing professionalism includes high ethical standards within a nurse’s behavioral standards to ensure patient safety and quality management [29,30]. As nursing professionalism improves, the ethical responsibility and awareness of patient privacy protection also increase. In the case of nursing students, clinical practice experience is an important environmental factor that establishes nursing professionalism [31]. To increase the perception of patient privacy among nursing students, nurses need to act as role models for patient privacy protection in addition to ensure correct training for nurses and nursing students to raise awareness of patient privacy protection in teaching institutions.

One notable finding of this study was that nursing informatics competency mediates the positive association between nursing professionalism and perception of patient privacy protection, supporting hypothesis H2. In other words, nursing professionalism can increase nursing informatics competency and lead to an increased perception of patient privacy protection. However, there were limitations in comparing the results of this study with other studies that confirmed the relationship between nursing professionalism and informatics competency due to the scarcity of such studies. Previous studies found that nursing professionalism showed a significant positive correlation with patient information management and information literacy (the ability to collect, interpret, and judge necessary information correctly), which is a similar concept to a sub-concept of nursing informatics competency [5,6,7,8,9,10,11,12,13,14,15,16,17,18,19,20,21,22,23,24,25,26,27,28,29,30,31,32]. Nursing professionalism includes practical competency along with ethical standards in a rapidly changing medical environment [29,30]. Recent nursing practice includes the concept of information management, such as electronic medical records (EMR) management [33]. As nursing professionalism improves, job performance in nursing practice increases [34], which can be interpreted as increasing nursing informatics competency. Furthermore, the role of nursing, a sub-concept of nursing professionalism, refers to the behavior that nurses are expected to have in nursing practice [13]. Nursing informatics competency is a core competency of nurses and nursing students [35], and this is thought to be the result of recent nursing students receiving information education in nursing education using EMR, and the recognition that informatics-related skills are needed when making important decisions for optimal patient outcomes.

This study also found that nursing informatics competency can predict perception of patient privacy protection. This finding is consistent with a previous study that found that nursing students’ information literacy as one of the concepts of nursing informatics competency influences patient privacy protection [8]. Recently, ethics, information protection, and various information and communication technology use capabilities have been added to the sub-factors in nursing informatics competency [36]. Moreover, among the technology informatics guiding education reform (TIGER) informatics competencies, ethical management of health information is reported as an important informatics competency in the fields of clinical nursing and inter-professional coordination nursing [37].

In this study, patient information management, which is one of the sub-concepts of the tool measuring nursing informatics competency, also included questions about patient privacy and confidentiality. In this regard, this study showed that nursing informatics competency is affected by nursing professionalism, which is an ethical criterion for collecting, interpreting, and using patient privacy. As nursing professionalism increases, it is considered to have an increasingly positive effect on the ethical management of health information, which was included in nursing informatics competency, consequently raising awareness that patient privacy should be protected. In other words, when nursing students have high professional beliefs and ethics in handling patient privacy, their ability to handle and manage information increases, leading to an increase in the awareness of patient privacy protection. Thus, to raise perception of patient privacy protection among nursing students, it is suggested that schools and hospitals establish and implement systematic theoretical and clinical practice education programs to nurture desirable nursing professionalism among the prospective nurses based on ethical awareness, thereby cultivating nursing informatics competency. Along with studies to confirm the relationship between nursing professionalism and informatics competency, a future study to confirm the effect of nursing job performance on the relationship between the two is also suggested, as nursing professionalism may vary according to socio-cultural backgrounds.

This study had several limitations. First, a cross-sectional design limited the interpretation of causality. Second, the study has limited generalizability because data collection was only conducted on a specific sample of nursing students. Moreover, the scales used in this study were developed or modified for adaptability to the socio-cultural context in Korea. Third, since the responses were made through an online self-report questionnaire, the participants may have exaggerated or reduced their capabilities and perceptions as per their understanding.

## 5. Conclusions

This study was conducted to examine the level of nursing professionalism, nursing informatics, and perception of patient privacy protection. The mediating effect of nursing informatics competency on the relationship between nursing professionalism and perception of patient privacy protection was also examined. The results show that nursing professionalism is positively associated with nursing students’ perception of patient privacy protection. Furthermore, nursing informatics competency has mediating effects on the relationship between nursing professionalism and the perception of patient privacy protection. The results supported all of the study’s hypotheses.

As major users of clinical information systems, nurses occupy the largest portion of the medical workforce. Nursing professionalism and nursing informatics competency are two essential elements that should be present in nurses for them to be able to provide professional and high-quality nursing care, including patient privacy protection [5,38]. This should be further developed in the nursing curriculum. Our findings have significant implications considering the globally increasing rates of exposure to patient information. Nursing professionalism and competence in nursing informatics determine the perception of patient privacy protection. The mediating role of nursing informatics competency has implications for the development of curricula in nursing education that aim to improve nursing students’ competence in nursing informatics and increase their perception of patient privacy protection.

## Figures and Tables

**Table 1:** General characteristics and patient privacy protection education (N = 242).

Variable	N (%) or Mean ± SD	Range
Demographic (*n* = 242)		
Age	23.20 ± 1.283	22~29
Sex		
Male	29 (12.0)	
Female	213 (88.0)	
Grade		
3rd year student (Junior)	91 (37.6)	
4th year student (Senior)	151 (62.4)	
Religion		
Yes	70 (28.9)	
No	172 (71.1)	
Need for Patient privacy protection-related education		
Yes	221 (91.3)	
No or don’t know	21 (8.7)	
Education of Patient privacy protection (*n* = 139)		
Number		
1~2 times	92 (38.0)	
3~4 times	34 (14.0)	
5 times or more	13 (14.0)	
Location		
University	118 (52.4)	
Clinical practice hospital	107 (47.5)	
Time		
1 h or less	134 (97.9)	
1 h or more	5 (2.1)	

**Table 2:** Descriptive statistics and correlation among the main variables.

	Mean ± SD	Range	1	2	3
1. Nursing professionalism	105.28 ± 12.86	56–140	1		
2. Nursing informatics competency	92.76 ± 11.34	54–121	0.236 **	1	
3. Perception of Patient privacy protection	99.16 ± 9.39	70–115	0.307 **	0.289 **	1

** *p* < 0.001.

**Table 3:** Mediating effect of nursing informatics competency on the relationship between nursing professionalism and perception of patient privacy protection.

Outcome	Predictors	*B*	SE	*P*	LLCI	ULCI
Nursing informatics competency	Constant	99.08	17.43	<0.001	64.74	133.42
	Nursing professionalism	0.18	0.05	0.001	0.07	0.30
	*R*^2^ = 0.076, *F* = 3.256, *p* = 0.004					
Perception of Patient privacy protection	Constant	81.69	13.17	<0.001	55.73	107.65
	Nursing professionalism	0.09	0.04	0.021	0.01	0.17
	Nursing informatics competency	0.18	0.04	<0.001	0.09	0.27
	*R*^2^ = 0.326, *F* = 16.180, *p* < 0.001					

Covariate variables = age, gender, grade, religion, subjective perception of the need of patient privacy protection education, and education of patient privacy protection-related variables; LLCI = low limit confidence interval; ULCI = upper limit confidence interval.

## Data Availability

Not applicable.
